# Artificial Intelligence in Virus Detection: From Metagenomic Discovery to Clinical Decision Support and Population Surveillance

**DOI:** 10.3390/biotech15040090

**Published:** 2026-10-10

**Authors:** Georgia Liakopoulou, Aristotle G. Koutsiaris

**Affiliations:** Medical Informatics and Biomedical Imaging (MIBI) Laboratory, Faculty of Medicine, School of Health Sciences, University of Thessaly, Biopolis Campus, 41500 Larissa, Greece; geliakopoulou@uth.gr

**Keywords:** artificial intelligence, mNGS, bioinformatics, virus, pathogen, detection, clinical decision support, epidemiology, surveillance, outbreak

## Abstract

Artificial intelligence (AI) is increasingly central to modern virus detection, particularly as metagenomic next-generation sequencing (mNGS) expands diagnostic and surveillance capabilities beyond targeted assays. This paper reviews how AI supports virus detection across three interconnected levels. The metagenomic layer forms the technical core of the review, while the clinical and population sections examine how sequence-level AI outputs are translated into patient-level and public-health decision support. At the metagenomic level, AI addresses key bottlenecks caused by fragmented assemblies and weak homology signals. Foundation-model approaches such as ViraLM leverage pretrained genome representations to improve viral contig discrimination under short-contig conditions, whereas LucaProt integrates sequence- and structure-informed learning to detect highly divergent RNA viruses via RdRP (RNA-dependent RNA polymerase)-focused discovery. At the clinical level, targeted molecular tests remain effective when the suspected pathogen is known, while mNGS provides a complementary, hypothesis-free approach that can broaden etiological investigation in complex infections when laboratories apply appropriate quality control and clinicians interpret results in context. At the population level, early warning systems combine heterogeneous data sources (epidemiological, open-source, digital, environmental, and wastewater signals) with AI methods to enhance outbreak detection and forecasting. Across all levels, robust contamination control, transparent reporting, external validation, and standardized interpretation criteria determine real-world value. Overall, hybrid pipelines that integrate molecular evidence, clinical metadata, and population-scale surveillance within governed, reproducible workflows are likely to provide the greatest impact.

## 1. Introduction

Metagenomic next-generation sequencing (mNGS) has expanded virus detection beyond targeted assays by enabling broad, hypothesis-free analysis of nucleic acids from clinical and environmental samples [1,2]. Its diagnostic value nevertheless depends on the interpretation of sequencing findings within the relevant laboratory and clinical context.

Despite its broad detection capacity, mNGS produces large, heterogeneous datasets affected by host background, low-abundance viral reads, sequencing noise, contamination, fragmented assemblies, and incomplete reference databases [1,3]. These limitations become particularly important for novel or highly divergent viruses, for which conventional homology-based approaches may have reduced sensitivity [3]. Researchers increasingly apply artificial intelligence (AI) to improve sequence filtering, classification, viral discovery, and interpretation across these complex workflows [3,4,5].

Recent approaches illustrate this shift from conventional bioinformatics toward representation learning and deep learning. ViraLM uses a pretrained genome foundation model to support viral contig identification, whereas LucaProt combines sequence- and structure-informed learning to detect highly divergent RNA-dependent RNA polymerase (RdRP) proteins [3,5]. At the same time, translation into clinical and public-health practice remains limited by contamination control, validation, interpretability, reporting standards, and the need to integrate computational outputs with laboratory and clinical evidence [1,6,7,8].

Key terminology used throughout this review is defined as follows. A foundation model is a model pretrained on large sequence corpora to learn generalizable representations and then adapted to a specific downstream task, such as viral contig classification. A contig is a contiguous nucleotide sequence reconstructed from overlapping sequencing reads. The term viral “dark matter” is used in this review as a broad metaphor for uncharacterized or poorly characterized viral sequence diversity that remains difficult to recognize with available reference-based methods. Where a narrower meaning is intended, this is stated explicitly; for example, in the LucaProt discussion, the term refers specifically to previously unrecognized viral RdRP sequence space. RNA-dependent RNA polymerase (RdRP) is a conserved viral enzyme that serves as a hallmark target for RNA-virus discovery. “Structure-informed” learning refers to approaches that incorporate predicted protein-structure features in addition to primary sequence information. Finally, “hypothesis-free” mNGS denotes sequencing that does not require a single predefined pathogen target, although interpretation still depends on analytical pipelines, reference resources, and validation.

Accordingly, this review examines AI-assisted virus detection across three connected levels: metagenomic analysis, clinical interpretation, and population surveillance. The metagenomic level provides the technical core because this is where sequence-level AI models operate most directly, whereas the clinical and population sections focus on translation, decision support, validation, and deployment. Section 2 introduces virus detection from patient specimens and the mNGS workflow, Section 3 examines five AI contributions across the bioinformatics pipeline, Section 4 provides an integrative synthesis of benefits, limitations, risks, and deployment requirements, Section 5 considers surveillance and early-warning applications, and Section 6 presents the main conclusions.

## 2. Clinical Level: AI-Enabled Virus Detection from Patient Specimens

At the clinical level, the central question is how laboratory-generated mNGS data can be converted into evidence that supports patient-level diagnosis and decision-making. This section therefore begins with the specimen-to-sequencing workflow and then shows how computational outputs must remain connected to clinical context, conventional diagnostics, and reporting requirements.

In clinical settings, virus detection relies primarily on patient specimens and accompanying clinical information, allowing laboratories to interpret molecular findings within the relevant clinical context [1]. Conventional laboratory diagnosis uses targeted assays most effectively when the suspected virus is known and the protocol targets a specific analyte [1]. Contemporary clinical microbiology increasingly uses metagenomic next-generation sequencing (mNGS) as a complementary diagnostic approach because it can detect a broad range of pathogens from the same sample and does not require a single predefined etiological target [1,2]. Unlike targeted tests such as PCR, which require preselection of specific agents, mNGS broadens the diagnostic search space, although its clinical usefulness still depends on rigorous interpretation and integration with conventional diagnostics [1,2].

Metagenomic sequencing analyzes samples that may contain multiple organisms and therefore typically requires noise reduction, host-read subtraction, and taxonomic classification [1]. Its relatively unbiased design supports broader pathogen detection, but it also increases interpretive complexity because samples may contain low-abundance viral reads, contaminants, or sequences absent from current databases [1]. At the interpretation stage, computational workflows can combine molecular findings with clinical variables and other contextual evidence to support clinically meaningful reporting [1,6,7].

Clinical studies have evaluated mNGS as a complementary tool for etiological investigation in complex infections, including pulmonary infections, when clinicians interpret sequencing results alongside conventional diagnostics and clinical assessment [2]. In severe pneumonia, recent work shows that mNGS can broaden pathogen identification and strengthen etiological investigation in diagnostically complex cases [9].

Clinical interpretation requires clear reporting procedures that connect sequencing findings with the patient’s presentation, history, treatment, and relevant risk factors [1]. Typical workflows begin with read-level quality control and filtering, followed by host-read subtraction and taxonomic profiling; many also assemble reads into contigs to improve downstream characterization and reduce reliance on single-read signals [1,4].

**Figure 1:**
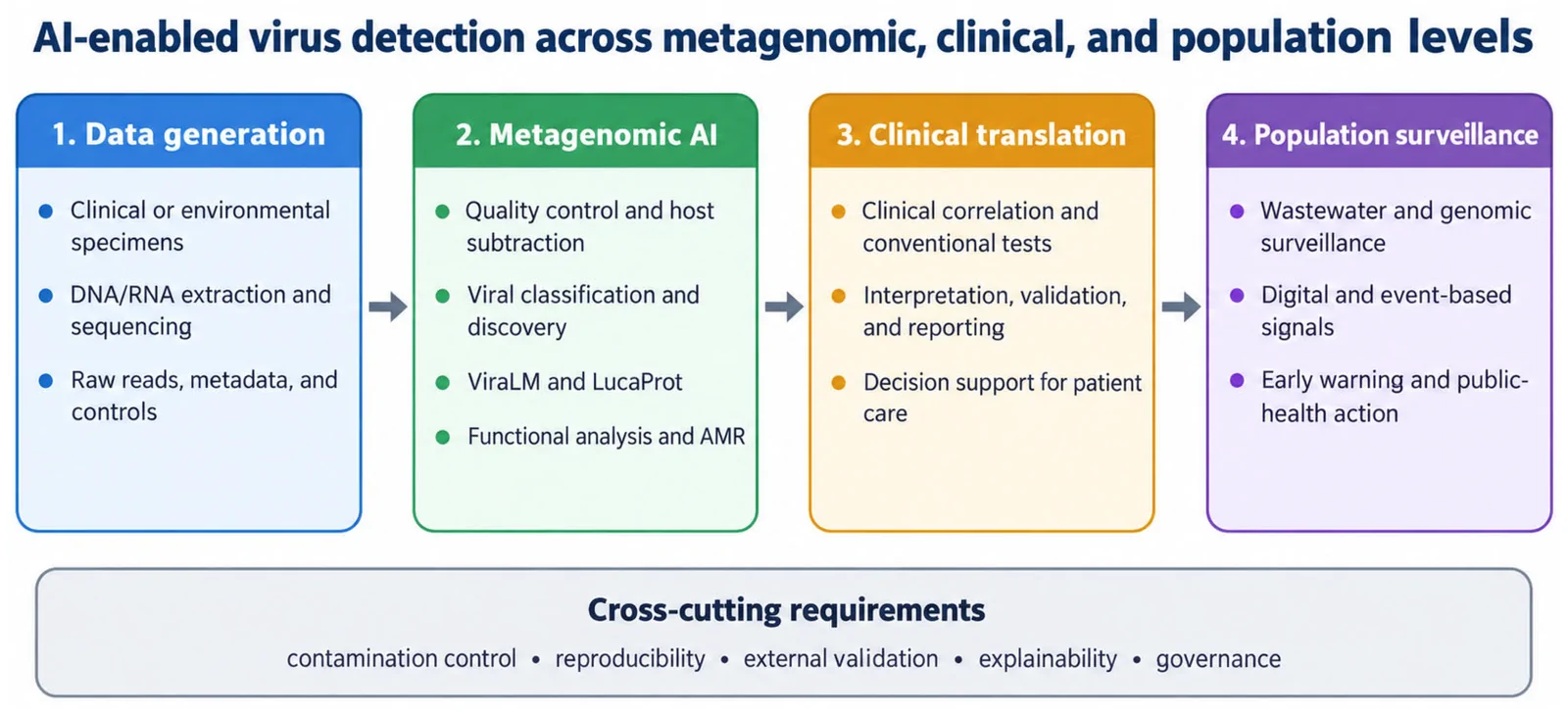
Integrated framework for AI-enabled virus detection across metagenomic, clinical, and population levels. The workflow begins with laboratory or environmental specimen processing and sequencing-data generation, then proceeds through AI-assisted metagenomic analysis, including quality control, host subtraction, viral classification/discovery, and functional analysis. These outputs can support patient-level interpretation and reporting or feed into population-level surveillance and early-warning activities. Cross-cutting requirements include contamination control, reproducibility, external validation, explainability, and governance.

Applied bioinformatics workflows can combine conventional mNGS processing with modules for pathogen detection, validation, and variant/SNP discovery, converting sequencing data into reproducible viral findings [4]. The laboratory stages that generate these data include sample collection, DNA/RNA extraction, library construction, and sequencing; Figure 1 places these stages within the broader pathway from data generation to metagenomic analysis, clinical interpretation, and population surveillance.

**Figure 2:**
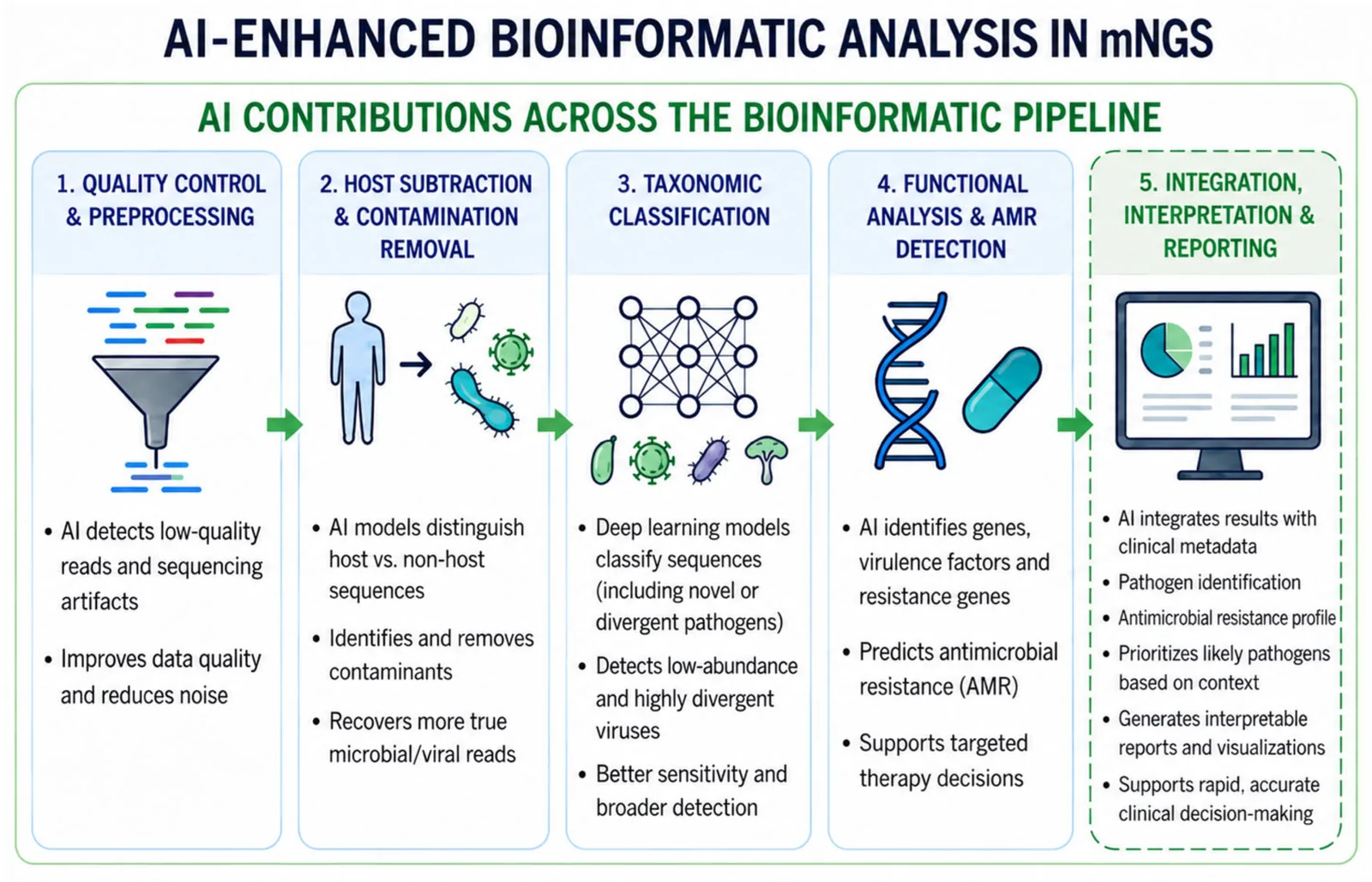
High-resolution overview of AI-enhanced contributions across the mNGS bioinformatic pipeline. The figure summarizes five linked contribution areas—quality control and preprocessing, host subtraction and contamination removal, taxonomic classification, functional analysis and AMR detection, and integration, interpretation, and reporting—showing how AI supports the transition from raw sequencing data and metadata to clinically interpretable outputs. These contribution areas are presented as connected elements of a sequential analytical workflow rather than independent tasks.

Figure 1 provides the overarching framework linking metagenomic analysis with clinical interpretation and population surveillance, whereas Figure 2 provides a closer view of the five AI contribution areas within the metagenomic bioinformatics component. To improve viral-sequence recovery and characterization, mNGS workflows increasingly incorporate AI-assisted operations across this bioinformatic pipeline.

AI can improve mNGS most directly during bioinformatic analysis, where raw reads must be transformed into interpretable evidence despite noise, mixed genomes, contamination risk, and low-abundance pathogens [1,6,7]. Figure 2 summarizes the five AI contribution areas examined in this section, from quality control and host subtraction to taxonomic classification, functional analysis, and integrated reporting.

The clinical workflow described above depends on the quality of the computational analysis that follows sequencing. The next section therefore moves from the patient-specimen context to the metagenomic level and examines, in workflow order, where AI can alter the reliability and interpretability of viral detection.

Beyond individual diagnostic case series, the clinical evidence base for mNGS now includes prospective and multicenter evaluations across several infectious-disease settings, although studies remain heterogeneous in patient selection, comparator testing, endpoints, and the extent to which sequencing results change management [1]. This distinction is important because diagnostic yield alone does not establish clinical utility: a result becomes clinically useful when it changes diagnosis, antimicrobial treatment, isolation decisions, or the need for additional testing. Clinical decision support should therefore do more than rank detected organisms. It should combine sequence confidence, abundance and coverage, control behavior, specimen characteristics, conventional microbiology, and the patient’s presentation into a transparent assessment that clinicians can review [6,7]. Routine adoption is also influenced by assay validation, reproducibility, quality-management systems, reporting standards, turnaround time, cost and reimbursement, and jurisdiction-specific accreditation or regulatory pathways [1,7]. Accordingly, the clinical translation of AI-assisted mNGS depends not only on analytical performance but also on validated workflow integration and accountable interpretation.

## 3. Metagenomic Level: Five AI Contributions Across the Bioinformatics Pipeline

At the metagenomic level, AI contributes across five sequential areas of the bioinformatics workflow: (1) quality control and preprocessing, (2) host subtraction and contamination removal, (3) taxonomic classification, (4) functional analysis including antimicrobial-resistance detection, and (5) integration, interpretation, and reporting. These five areas are discussed in Section 3.1, Section 3.2, Section 3.3, Section 3.4 and Section 3.5, respectively, and are presented in workflow order so that each stage builds on the output of the preceding one. ViraLM and LucaProt are discussed within the taxonomic-classification stage as representative AI approaches that address specific limitations of conventional viral identification and discovery.

### 3.1. Quality Control & Preprocessing

Quality control and preprocessing are essential in clinical mNGS because sequencing artifacts, low-quality bases, and low-complexity reads can propagate through the workflow and inflate false positives, especially in low-biomass specimens where microbial signal is weak relative to host background [1]. In practice, AI and machine learning (ML) can strengthen this stage by supporting robust filtering and prioritization of informative reads, which becomes increasingly important when downstream analyses must remain sensitive to low-abundance pathogens while controlling noise [1,4].

Preprocessing decisions also affect the performance of downstream AI-based classification modules, because models such as ViraLM operate on contigs or fragments whose statistical properties can be distorted by upstream noise and sequencing artifacts [5]. Similarly, LucaProt’s RdRP (RNA-dependent RNA polymerase)-focused discovery is most reliable when low-quality or contaminant-derived sequences are minimized early, since false signals at the input stage can mislead structure- and sequence-aware prediction modules [3].

### 3.2. Host Subtraction & Contamination Removal

Machine learning can support host subtraction and decontamination by learning patterns that distinguish host, contaminant, and non-host signals when simple thresholds are insufficient [1]. Its usefulness at this stage depends on well-characterized controls and on preprocessing choices that do not inadvertently remove low-abundance pathogen sequences.

Host subtraction is a core requirement in clinical mNGS because host-derived nucleic acids can dominate sequencing output in common specimen types (e.g., plasma, CSF, and BALF), substantially diluting microbial signal and reducing sensitivity in low-biomass infections [1]. Several host-depletion strategies can improve the pathogen signal-to-noise ratio, including chemical lysis followed by DNase digestion and CRISPR-Cas9-based depletion approaches such as Depletion of Abundant Sequences by Hybridization (DASH) [10]. However, host depletion also introduces trade-offs: multilaboratory evidence shows that depletion procedures can reduce microbial detection in low-biomass samples and therefore increase the risk of false negatives [11]. The balance between host depletion and microbial recovery should therefore be validated for the specimen type and workflow in use.

Contamination control is equally critical because reagent- and laboratory-derived DNA (“kitome”) can introduce spurious reads, particularly in low-biomass samples, and can materially distort downstream classification if not managed carefully [12,13]. In addition, cross-contamination during extraction—especially in plate-based workflows—can cause well-to-well carryover and alter apparent microbial profiles, which strengthens the case for contamination-aware handling and analysis [1,14]. As a result, recommended safeguards include no-template controls, extraction blanks, standardized mock communities, and internal spike-in controls to benchmark sensitivity and inter-run variability and to support defensible filtering [1].

From an AI perspective, host subtraction and contamination removal are enabling conditions for reliable downstream modeling, because classification models can amplify contaminant signals if preprocessing and controls are weak [1]. Therefore, AI-assisted workflows should be embedded in pipelines that explicitly document host-removal steps, contamination controls, and filtering thresholds, so that model outputs remain reproducible and clinically interpretable rather than functioning as stand-alone “black box” calls [1,8].

### 3.3. Taxonomic Classification

Downstream, deep learning–based sequence representations can improve taxonomic classification when homology is weak, which is especially relevant for short contigs and divergent viral sequences that may be missed by alignment-only approaches [5]. Such models can be integrated into pipelines to prioritize likely viral contigs for further review and validation, helping analysts focus on plausible candidates while reducing false positives driven by ambiguous matches [1,5].

Recent AI approaches for detecting viral contigs in metagenomic datasets use pretrained sequence representations to improve discrimination between viral and non-viral sequences [5]. Foundation models are particularly relevant because many real-world datasets contain short and fragmented contigs that challenge traditional homology-based strategies [5].

Reference databases underpin sequence-based virus detection and taxonomic assignment. NCBI resources aggregate viral nucleotide and protein records and provide dedicated tools for viral sequence retrieval and analysis [15]. Domain-focused resources such as the Virus Pathogen Resource (ViPR) add curated viral annotations and analysis tools [16], while BV-BRC combines bacterial and viral datasets with reproducible bioinformatic services [17]. GISAID supports outbreak genomics by providing large viral genome collections with associated metadata, particularly for influenza and SARS-CoV-2 [18].

For metagenomic discovery, specialized catalogs of uncultivated viral sequences such as IMG/VR provide viral genome fragments derived from metagenomes and support downstream analysis beyond classical homology searches [19]. Virus naming and higher-level organization are supported by the International Committee on Taxonomy of Viruses (ICTV), which provides the official taxonomy framework [20]. When likely host range is relevant, Virus-Host DB links viral genomes with host taxonomy and supports host-assignment tasks [21].

A central obstacle in metagenomic virus discovery is that many datasets contain short and fragmented contigs, while a substantial fraction of viral diversity is not represented in reference databases, limiting the sensitivity of purely homology-based approaches [3,5]. Under low sequence similarity, standard alignment-based methods can fail to recognize highly divergent viruses, and even when a virus is present, the recovered contigs may not contain enough recognizable similarity for confident classification [3,5]. This constraint motivates AI approaches that learn representations capturing broader statistical and functional signatures of viral sequences rather than relying exclusively on direct similarity to known references [5]. Large-scale pretraining allows the model to encode generalizable genomic patterns [5]. This can improve performance when labeled training data are incomplete, contigs are short, or target sequences differ substantially from those represented in curated references. This focus is practically important because fragmented contigs are a common output of metagenomic assembly and are frequently responsible for reduced sensitivity in traditional pipelines that depend heavily on local alignment evidence [5].

In this context, two recent directions illustrate how AI can extend metagenomic detection and discovery, namely foundation-model–based contig classification as exemplified by ViraLM and structure-informed deep learning for divergent RNA virus discovery as exemplified by LucaProt [3,5].

#### 3.3.1. ViraLM

**Figure 3:**
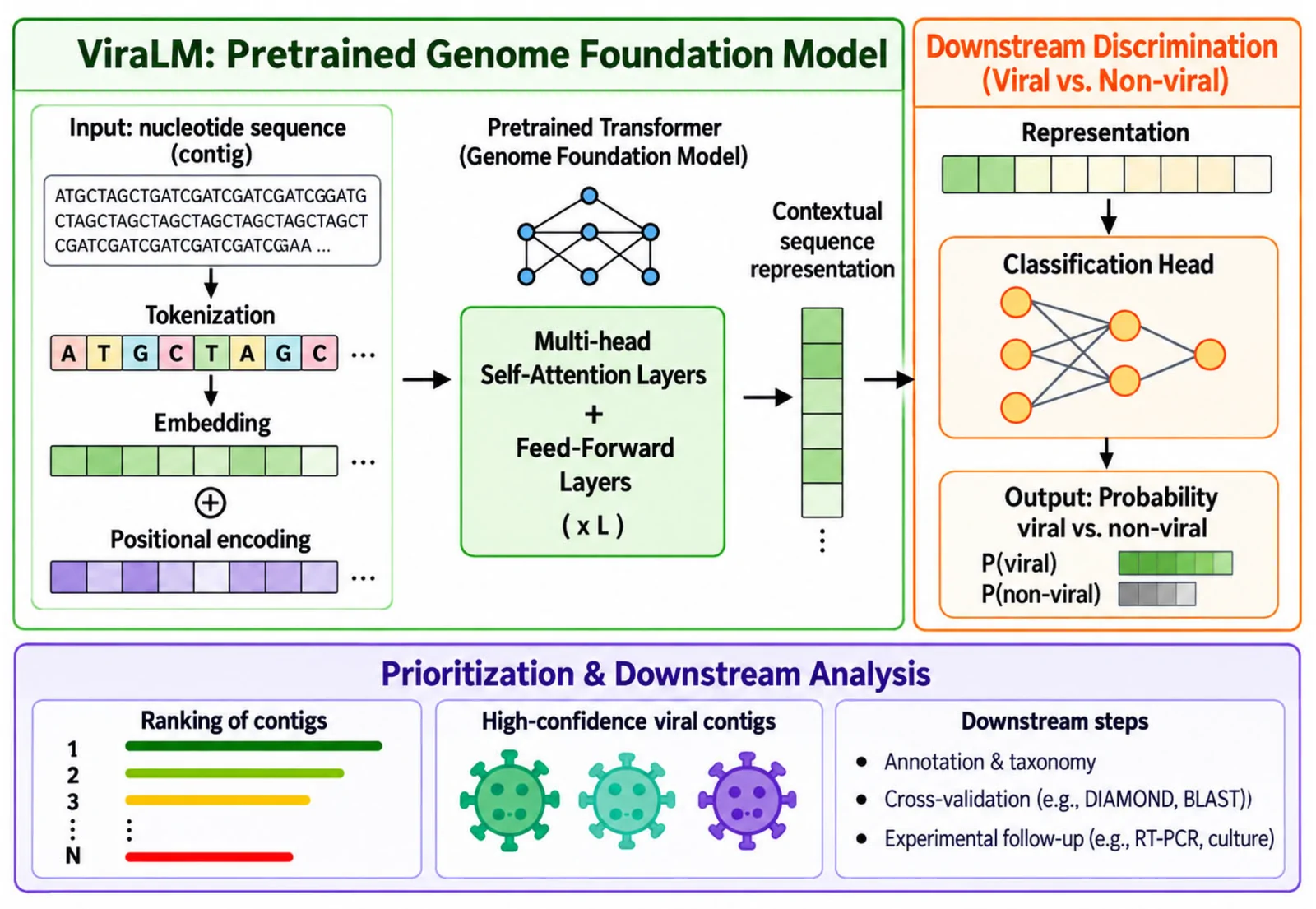
Schematic overview of the ViraLM workflow for viral contig identification. Input nucleotide sequences are tokenized using Byte-Pair Encoding and processed by the pretrained DNABERT-2 genome foundation model to obtain contextual sequence representations. A task-specific binary classification head estimates the probability that a sequence is viral. Candidate viral contigs can then be prioritized for downstream annotation, cross-validation, and experimental follow-up (adapted conceptually from [5]).

ViraLM uses the pretrained DNABERT-2 genome foundation model as its backbone to learn informative nucleotide-sequence representations and distinguish viral from non-viral contigs [5]. The approach transfers representations learned during self-supervised pretraining to the downstream virus-identification task. In ViraLM, input sequences are tokenized using the DNABERT-2 Byte-Pair Encoding (BPE) scheme, processed by the pretrained transformer backbone, and then fine-tuned with labeled viral and non-viral examples through a binary classification objective (Figure 3) [5].

The ViraLM architecture comprises three main components (Figure 3): a Byte-Pair Encoding block, a multi-layer Transformer block initialized from DNABERT-2, and a binary classifier for virus identification [5]. During prediction, an input sequence is divided into non-overlapping 2 kb fragments. Each fragment is tokenized and assigned a viral-probability score between 0 and 1, and fragment scores from the same sequence are averaged to obtain the final sequence-level prediction. By default, a final score greater than 0.5 is classified as viral [5]. The positive training set contained 49,929 high-quality viral genomes, while the negative set included sequences from organisms and genetic elements that may be confused with viruses in metagenomic data, including bacteria, archaea, plasmids, and eukaryotic hosts or vectors [5]. Benchmarking showed improved performance on short contigs, including a reported 22% improvement in F1-score under short-contig conditions [5].

From an operational standpoint, ViraLM-like models can be placed after assembly and basic filtering to prioritize candidate viral contigs for annotation, cross-validation, and targeted experimental follow-up [5]. Clinical mNGS studies nevertheless show that metagenomic calls require cautious interpretation. False-positive signals can arise from contamination, low-complexity artifacts, or ambiguous matches, so model predictions should remain linked to transparent reporting and validation workflows rather than being treated as stand-alone proof of infection [1].

From a critical perspective, ViraLM should not be interpreted as a clinically validated diagnostic system. The ViraLM study [5] evaluated the model on benchmark datasets and on three real metagenomic datasets and reported a 22% improvement in F1-score for short contigs, but the study did not establish prospective false-positive or false-negative rates in routine clinical specimens. The authors also note two practical limitations: ViraLM does not provide taxonomic or gene-annotation output, and transformer-based processing becomes computationally challenging for very long nucleotide sequences [5]. These limitations mean that ViraLM is best positioned as a prioritization tool for candidate viral contigs, followed by annotation, orthogonal bioinformatic checks, and, where clinically relevant, laboratory confirmation.

Deployment feasibility also depends on local infrastructure because transformer-based sequence analysis benefits from GPU acceleration and can become resource-intensive as dataset size increases. ViraLM predictions may also be affected by dataset composition, sequence length, contamination, and domain shift between research datasets and clinical material. Benchmark superiority should therefore not be equated with routine diagnostic utility until external, multicenter validation establishes performance across specimen types, sequencing platforms, and laboratories.

#### 3.3.2. LucaProt

LucaProt addresses a related but distinct challenge: the discovery of highly divergent RNA viruses in metatranscriptomic data when conventional homology searches lose sensitivity because sequence similarity is very low [3]. Rather than functioning as a general RNA-virus classifier, LucaProt was developed to identify divergent viral RNA-dependent RNA polymerase (RdRP) sequences by integrating amino-acid sequence information with predicted structural representations. Applied to 10,487 metatranscriptomes, the approach contributed to the identification of 161,979 putative RNA virus species and 180 RNA virus supergroups [3].

LucaProt was designed to detect divergent RNA-dependent RNA polymerase (RdRP) sequences by integrating primary sequence information with predicted structural signals [3]. This design is important because structural and functional constraints may remain detectable even when nucleotide or amino-acid similarity becomes too low for reliable alignment-based identification. LucaProt therefore focuses on RdRP as a conserved hallmark gene of RNA viruses and uses deep learning to capture both local and long-range sequence dependencies. The LucaProt study [3] describes the model as a transformer-based approach for identifying the previously unrecognized viral RdRP sequence space, used here as a specific instance of viral “dark matter” when conventional homology searches lose sensitivity. The authors combined LucaProt with a more conventional pipeline (ClstrSearch), and the merged strategy supported large-scale RdRP-based discovery across thousands of metatranscriptomes.

**Figure 4:**
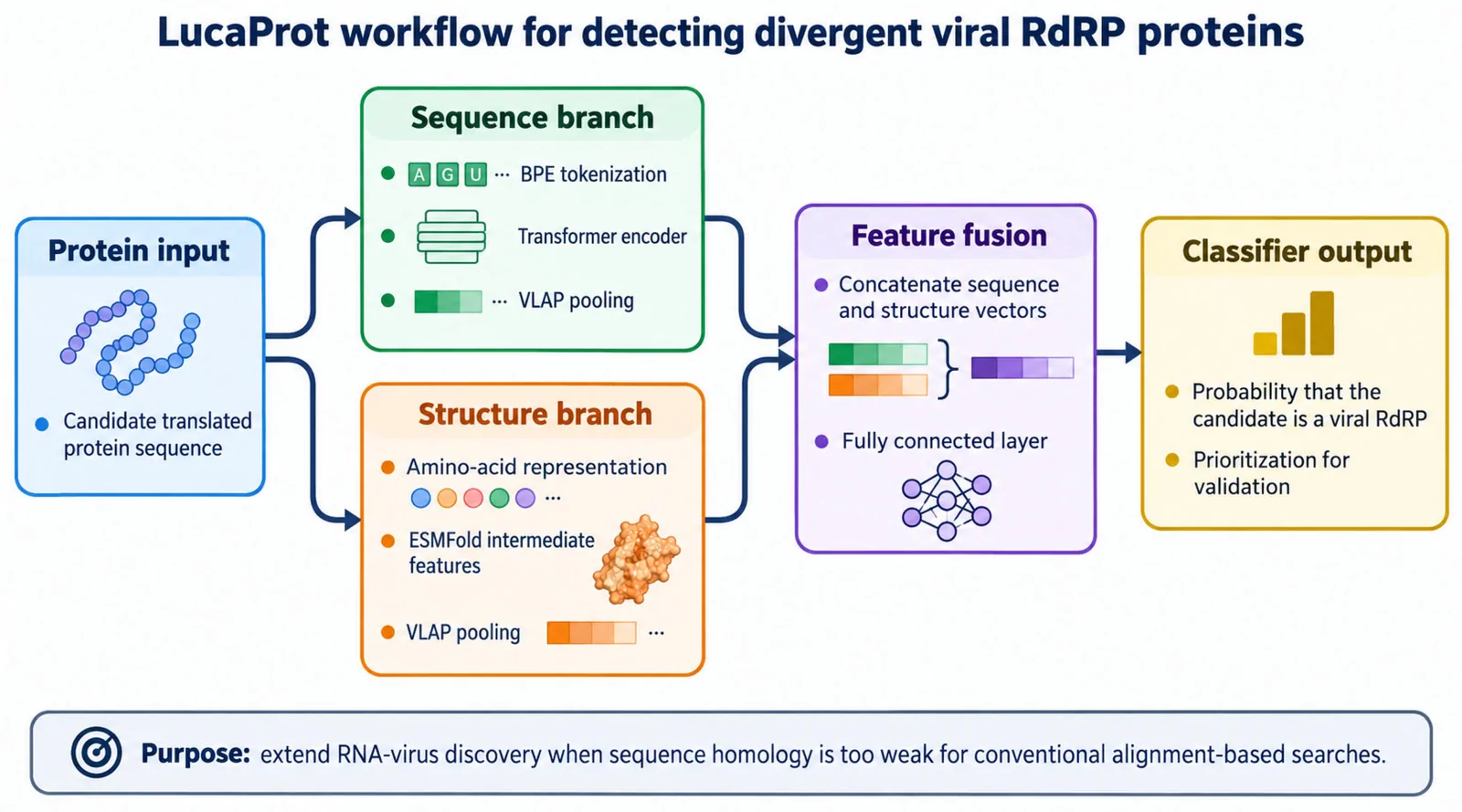
Schematic overview of the LucaProt workflow for detecting divergent viral RdRP proteins. Candidate translated protein sequences are processed through two complementary branches: a sequence branch using BPE tokenization, Transformer encoding, and Value-Level Attention Pooling, and a structure-informed branch based on ESMFold-derived representations followed by the same pooling strategy. The pooled sequence and structure vectors are concatenated and passed through a fully connected classifier to estimate the probability that the candidate is a viral RdRP. High-probability candidates are then prioritized for downstream validation and virus-discovery analyses (adapted conceptually from [3]).

At the model level, LucaProt uses two complementary feature branches (Figure 4). The sequence branch applies BPE-based subword tokenization and a Transformer encoder to protein sequences, followed by Value-Level Attention Pooling (VLAP). In parallel, the structure-informed branch uses ESMFold-derived intermediate representations to capture predicted structural information and also applies VLAP. The resulting sequence and structure vectors are then combined for downstream classification [3].

After pooling, LucaProt concatenates the sequence-derived and structure-derived feature vectors and passes the fused representation through a fully connected classification layer (Figure 4). The output is a probability that the candidate protein is a viral RdRP, with 0.5 used as the binary classification threshold in the reported implementation. The model-building dataset contained 235,413 samples, including 5979 known viral RdRPs as positive examples and 229,434 negative examples drawn from non-viral RdRPs and other protein categories [3]. The available LucaProt resources also document a 20,000-token subword vocabulary and value-attention pooling for both sequence and structural representations.

The reported application of LucaProt across large metatranscriptomic datasets supports the view that extensive RNA viral diversity remains hidden in environmental and host-associated samples and that structure-aware learning can expand the detectable RNA virome beyond the limits of conventional reference-based screening [3]. In practical terms, LucaProt-like approaches can be used as targeted discovery modules within metatranscriptomic workflows to screen for hallmark genes, prioritize highly divergent candidates, and support downstream steps such as taxonomic placement and validation [3].

Taken together, recent work suggests that AI methods such as ViraLM and LucaProt are most impactful when integrated into well-controlled pipelines that combine representation learning with rigorous validation and reporting, thereby enabling both improved detection of fragmented viral signals and expanded discovery of highly divergent viral diversity [3,5].

LucaProt also requires cautious interpretation because its strongest evidence concerns large-scale RNA-virus discovery rather than routine diagnosis. The original LucaProt study [3] applied the model to 10,487 metatranscriptomes and reported 161,979 putative RNA virus species, with a subset subsequently confirmed by RT-PCR and RNA/DNA sequencing. This experimental confirmation supports the discovery framework, but it does not provide a patient-level clinical false-positive rate. The model therefore demonstrates discovery capability rather than validated diagnostic accuracy.

Practical deployment is further constrained by the structure-informed component. LucaProt combines transformer-based sequence encoding with ESMFold-derived structural representations, which increases computational complexity relative to simpler alignment- or sequence-only approaches [3]. This may be acceptable for research-scale virus discovery, where high-performance computing resources can be available, but routine clinical or public-health laboratories would additionally need evidence on turnaround time, hardware requirements, reproducibility, and performance under real-world contamination and low-biomass conditions. In its current form, LucaProt is therefore more appropriately viewed as a discovery and candidate-prioritization platform than as a stand-alone clinical diagnostic tool.

### 3.4. Functional Analysis & AMR Detection

Metagenomic analysis may also support identification of antimicrobial resistance (AMR) determinants through detection of resistance-associated genes, but clinical actionability depends on quality control, interpretation standards, and correlation with patient context [1]. AMR refers to the ability of microorganisms to withstand antimicrobial agents that would normally inhibit or eliminate them; in metagenomic workflows, this is assessed primarily through the detection and interpretation of resistance-associated genetic determinants [22,23].

Metagenomic AMR profiling depends heavily on which resistance gene reference database and detection models are used, because different databases vary in scope, curation strategy, ontology, and update frequency, which can change which genes are reported and how they are interpreted [22,23]. In this context, curated resources such as the Comprehensive Antibiotic Resistance Database (CARD) provide ontology-driven resistome annotation frameworks and gene detection models for AMR surveillance, while MEGARes 2.0 provides a structured resistance-gene catalog designed for high-throughput metagenomic classification [22,23]. Because AMR “hits” are ultimately database-dependent, AMR profiles should be reported with explicit mention of the reference database/version and the detection criteria, so that results are reproducible and comparable across studies and laboratories [22,23].

Beyond database choice, rapid time-to-result makes AMR profiling clinically and epidemiologically attractive, especially when laboratories use fast sequencing platforms directly on respiratory or other time-sensitive specimens [24]. For example, targeted nanopore sequencing can detect both pathogens and resistance genes in lower respiratory tract infection samples, illustrating the feasibility of accelerated workflows that combine molecular detection with resistance-gene evidence [24]. Clinical interpretation should nevertheless remain cautious because detection of a resistance-associated gene does not automatically establish phenotypic resistance; AMR profiles therefore provide genotypic evidence that supports, rather than replaces, clinical correlation and antimicrobial-stewardship decisions [1,24].

### 3.5. Integration & Interpretation Reporting

Integration and reporting are the point where mNGS outputs become clinically meaningful, because the pipeline must move from raw hits to an interpretable statement about plausible causality, confidence, and clinical relevance in the specific patient context [6]. This requires combining multiple evidence layers, including read/contig abundance patterns, genome/contig coverage, negative-control behavior, and consistency with clinical presentation, while explicitly documenting the decision rules used to determine what is reportable versus likely background or contamination [6,7]. As a result, recent discussions emphasize that clinical-grade reporting depends on standardized workflows and quality management practices (e.g., traceable controls and consistent thresholds) rather than treating a bioinformatics output as a stand-alone diagnostic conclusion [7].

Across AI-enabled metagenomic pipelines, reliability depends not only on model architecture but also on evaluation design, contamination control, and transparent reporting [1]. The clinical mNGS literature stresses that results should be interpreted with contextual evidence and standardized reporting procedures, since metagenomic signals can reflect low-abundance organisms or non-causal findings if not carefully evaluated [1]. A key integration challenge is reporting transparency: different pipelines, databases, and thresholds can produce materially different organism lists and confidence levels, which makes cross-study and cross-laboratory comparison difficult unless reporting is standardized and sufficiently detailed [25]. Recommendations for virome-scale reporting stress the need for clear communication of sample processing, analysis parameters, reference resources, and uncertainty, so that findings can be interpreted in evolutionary/ecological context and reused by others [25].

In clinical workflows, this translates into practical reporting elements such as the rationale for calling a detection, how low-abundance signals were handled, how controls were used to flag contamination, and what limitations remain for clinical action ability [6,7].

Integration and reporting also require external validation and workflow-level performance evidence, especially when an assay is intended for routine clinical decision-making rather than exploratory research [26]. Clinical validation studies of largely automated mNGS assays for agnostic detection of respiratory viral pathogens show why performance claims should rely on real clinical samples and transparent reporting of turnaround time, sensitivity/specificity trade-offs, and operational constraints [26]. When AI components support prioritization or interpretation, the same principle applies: model outputs are most defensible when they remain embedded in validated pipelines with explicit quality control, standardized reporting, and clinical correlation rather than functioning as opaque “black-box” conclusions [6,7,8].

Taken together, these five metagenomic stages show that AI performance is cumulative: weaknesses in preprocessing or contamination control can propagate into classification and reporting, whereas improvements at earlier stages can increase the reliability of downstream interpretation. The following section therefore shifts from individual tools to a cross-stage synthesis of the main benefits and trade-offs.

## 4. Integrative Synthesis: From Metagenomic Analysis to Clinical Use

The preceding sections describe individual workflow components; this section synthesizes how these components interact. The value of AI is not confined to a single classification step: improvements in preprocessing affect downstream sensitivity, stronger classification changes which candidates reach validation, and better integration determines whether a metagenomic signal becomes clinically actionable. The benefits summarized here should therefore be interpreted as workflow-level effects rather than isolated algorithmic advantages. To provide a more substantive synthesis of the literature, Table 1 compares representative AI-enabled approaches and explicitly separates analytical or algorithmic limitations from operational or deployment issues.

Table 1 shows that the main AI approaches considered in this review address different stages of the same evidence chain. Sequence-based models such as ViraLM and LucaProt primarily improve candidate identification or discovery, whereas clinical mNGS frameworks emphasize interpretation and reporting, and AI-enabled surveillance systems operate at the population level. Their outputs are therefore complementary rather than interchangeable: a high-probability viral contig, a putative RdRP, a clinical report, and a population alert represent different forms of evidence with different validation requirements. The table also distinguishes analytical or algorithmic limitations from operational or deployment issues to make these two constraint types easier to compare across applications.

Across these applications, potential gains in sensitivity, speed, and analytical breadth must be interpreted together with the risk of ambiguous findings, domain shift, computational burden, and limited external validation. This distinction is important because strong benchmark performance does not necessarily translate into reliable routine use. The practical value of AI therefore depends on how well each tool fits the surrounding laboratory, clinical, or public-health workflow and on whether its outputs can be independently verified.

These benefits must be balanced against several limitations. Higher analytical sensitivity may also increase the number of ambiguous or incidental findings, while reduced benchmark error does not guarantee equivalent performance after changes in specimen type, sequencing platform, prevalence, or laboratory workflow. For both ViraLM and LucaProt, the published evidence is substantially stronger for research and discovery settings than for prospective clinical deployment [3,5]. Accordingly, the most important evidence gap is not whether these models can detect difficult viral signals, but whether their performance remains calibrated, interpretable, and operationally useful when embedded in routine laboratory and public-health workflows.

Future evaluations should therefore report not only aggregate accuracy or F1-score but also externally validated false-positive and false-negative rates, calibration, computational requirements, turnaround time, and failure modes across independent datasets. Such information is necessary to distinguish promising algorithmic performance from clinically or operationally mature implementation.

### 4.1. Practical Examples of AI-Enabled Implementation

Several studies provide concrete examples of AI or AI-assisted approaches being applied to real sequencing data rather than only proposed as future possibilities. ViraLM was evaluated not only on benchmark datasets but also on three real metagenomic datasets, where it was used to prioritize viral contigs and showed improved performance on short contigs [5]. LucaProt was deployed at much larger discovery scale across 10,487 metatranscriptomes and identified 161,979 putative RNA virus species; a subset of candidates was subsequently confirmed experimentally [3]. These applications demonstrate practical use of AI for sequence prioritization and virus discovery, although neither study establishes routine clinical deployment.

A second example is the AI-enabled NGS pipeline developed for virus detection, validation, and SNP discovery [4]. The pipeline was tested on previously analyzed RNA-seq and whole-genome datasets containing influenza virus, SARS-CoV-2, an Enterococcus phage, and maize Iranian mosaic virus, illustrating how AI-assisted and conventional bioinformatic steps can be combined in an end-to-end workflow [4]. At the clinical end of the spectrum, automated mNGS assays have also undergone laboratory validation using real respiratory specimens, providing an operational model for how algorithmic analysis can be embedded within a controlled diagnostic workflow even when the analytical stack is not based on a single deep-learning model [26]. Together, these examples show that practical implementation already exists, but most evidence remains at the validation or research-deployment stage rather than routine clinical use.

### 4.2. Cross-Cutting AI Limitations and Risks

A broader limitation of AI in virology is the risk of over-reliance on models whose internal decision processes are difficult to interpret. Deep learning systems can assign high-confidence scores without providing a biologically transparent explanation for the prediction, particularly when they operate on complex sequence representations. In clinical and public-health settings, this “black-box” behavior can make it difficult to distinguish a biologically meaningful signal from a model-specific artifact. For this reason, AI outputs should remain traceable to underlying sequence evidence, quality-control metrics, reference information, and confirmatory laboratory findings rather than being treated as autonomous conclusions [8].

Algorithmic bias represents a second cross-cutting risk. Training datasets may over represent particular viral taxa, geographic regions, sequencing platforms, specimen types, or well-characterized outbreaks. Models trained under these conditions may perform less reliably on underrepresented populations or on data generated with different laboratory protocols. In metagenomic analysis this can appear as domain shift or systematic under-detection of poorly represented viral diversity; at the population level, similar biases can arise from unequal access to testing, sequencing, wastewater monitoring, or digital data streams [8,28,29,30]. External validation across independent laboratories, populations, and data-generating conditions is therefore necessary before broad deployment.

A third risk is automation bias: users may give disproportionate weight to an AI-generated classification or alert because it appears quantitative or technically sophisticated. This is particularly problematic when evidence is weak, contamination is present, or a model encounters data outside its training distribution. Human-in-the-loop review, explicit uncertainty reporting, predefined escalation criteria, and periodic post-deployment performance monitoring can reduce this risk. These safeguards are especially important when AI outputs influence patient management, outbreak investigation, or public-health resource allocation [1,8].

A further clinical risk is over-detection. Highly sensitive AI-assisted mNGS workflows may flag low-abundance organisms, contaminants, latent or non-causal sequences, or other findings that are technically detectable but not clinically actionable [1,6,7]. If such findings are reported without appropriate contextual filtering, they may trigger unnecessary confirmatory testing, additional consultations, patient concern, or avoidable use of clinical resources. Clinical reporting should therefore distinguish clearly between detected sequence evidence and findings judged to be clinically significant, using predefined reporting thresholds, negative and process controls, orthogonal confirmation where appropriate, and explicit communication of uncertainty [1,6,7,25,26]. Human review remains essential when a result is unexpected, weakly supported, or discordant with the clinical presentation.

Explainability can also support safer use of AI, but it should be applied with realistic expectations. Practical approaches may include attention-based visualizations, feature-attribution methods, or simpler surrogate models that help identify which sequence regions or learned features contributed most strongly to a prediction. Such methods can assist expert review, reveal possible artifacts, and make model behaviour easier to audit, but they do not by themselves establish biological causality or diagnostic validity. For clinical use, explainability is therefore most useful when combined with traceability to the underlying reads or contigs, quality-control metrics, reference evidence, confidence estimates, and confirmatory laboratory findings, rather than being treated as a substitute for validation [8].

### 4.3. Practical Deployment and Resource Requirements

Routine deployment also has resource implications that are not captured by accuracy metrics alone. AI-assisted mNGS requires sequencing infrastructure, data storage, maintained reference databases, version-controlled software, and personnel able to evaluate discordant or uncertain findings [1,7,26]. Transformer-based sequence models and structure-informed approaches can add computational requirements beyond simpler alignment-based screening, particularly when laboratories process large datasets or require rapid turnaround [3,5]. These requirements may limit implementation in settings without dedicated bioinformatics or high-performance computing support.

Deployment requirements also vary substantially by setting. A national reference laboratory may be able to support high-throughput sequencing, centralized validation, advanced computing, and national surveillance functions, whereas a regional public-health laboratory may prioritize interoperability, referral pathways, standardized workflows, and rapid escalation of unusual findings. Hospital clinical microbiology laboratories, by contrast, face stronger constraints related to turnaround time, integration with existing diagnostic services, clinical reporting, accreditation, and direct support of patient-management decisions [1,7,26].

Costs should therefore be considered at the workflow level rather than as the price of a single algorithm. Relevant burdens include sequencing and quality-control procedures, computational hardware, data storage, software maintenance, staff training, external validation, and confirmatory testing. The reviewed studies do not provide a standardized cost-effectiveness comparison across these components, so no firm conclusion can yet be drawn about economic superiority over existing diagnostic pathways. Future implementation studies should report resource use and turnaround time alongside diagnostic performance to determine where AI-assisted workflows provide practical value [1,7,26].

The analytical gains summarized above are most useful when they can be translated beyond a single sample or patient. The next section considers this population-level extension, focusing on how laboratory and metagenomic outputs can contribute to surveillance and early-warning systems.

## 5. Population Level: Detection, Surveillance, and Early Warning

The population level extends the same analytical chain beyond the individual patient. Viral sequences and candidate taxa generated by clinical or environmental mNGS can become inputs to genomic and wastewater surveillance, while population-level signals can in turn identify where additional sequencing or targeted investigation is needed. This creates a feedback loop in which metagenomic AI, clinical interpretation, and surveillance reinforce one another rather than functioning as separate domains.

The tools discussed earlier can contribute to this loop in different ways. ViraLM-like classifiers can help prioritize previously unrecognized viral contigs from large clinical or environmental sequencing datasets, whereas LucaProt-like approaches can extend RNA-virus discovery when conventional homology searches fail [3,5]. Once validated and taxonomically characterized, such outputs can expand reference resources, improve subsequent screening, and support genomic surveillance of emerging viral diversity. Conversely, signals detected through wastewater or event-based surveillance can guide where metagenomic sequencing and AI-assisted discovery should be intensified.

Population-level surveillance combines systematic epidemiological monitoring with additional data streams that may provide earlier or complementary signals [28,29]. AI is useful in this setting because it can integrate large, heterogeneous datasets and prioritize anomalies for human review. However, these outputs are only useful when bias, representativeness, and validation against established surveillance indicators are explicitly addressed [27,29].

Event-based surveillance uses open-source reports, media, and digital information streams to identify potential threats [30,31,32,33]. Systems such as WHO’s Epidemic Intelligence from Open Sources (EIOS) illustrate how automated collection and prioritization can support early detection, but candidate signals still require verification, calibration, and interpretation before public-health action is taken [30,31,32,33].

Digital surveillance can provide leading indicators from search behavior, online platforms, and social media, but such signals are vulnerable to media effects, selection bias, and spurious correlations [29,34]. Natural-language processing can convert unstructured reports into structured indicators, yet these signals should be treated as prompts for investigation rather than direct evidence of viral circulation [27,29,35]. Where feasible, targeted clinical or environmental sequencing can provide the molecular confirmation needed to connect an epidemiological signal to a specific pathogen or variant.

Wastewater-based surveillance provides a particularly direct connection to the metagenomic workflow because community samples can contain viral nucleic acids that are suitable for sequencing and downstream computational analysis [36,37,38]. Machine-learning methods are used for time-series modeling, trend forecasting, and covariate integration, whereas sequence-based AI can assist with the prioritization of novel or divergent viral signals. In a connected workflow, a wastewater anomaly can trigger deeper sequencing, AI-assisted classification or discovery, and subsequent validation before the signal is incorporated into population-level interpretation [36,37,38,39].

The evidence base for AI-enabled population surveillance also differs from that for patient-level diagnosis. Many surveillance studies evaluate retrospective forecasting, anomaly detection, or proof-of-concept models rather than direct effects on public-health outcomes [27]. Operational deployment therefore requires prospective assessment of timeliness, false-alert burden, representativeness, robustness to data drift, and whether an alert actually changes sampling, investigation, or response decisions. A useful public-health decision-support layer should combine genomic or wastewater signals with epidemiological baselines and transparent escalation thresholds, while preserving human review, traceability, and governance [27,28,29,36,37,38,39]. These requirements help distinguish an informative surveillance signal from an actionable early-warning system.

Accordingly, surveillance should be viewed as the downstream extension of the same evidence chain described earlier in this review. Laboratory sampling generates molecular data; metagenomic AI prioritizes and characterizes viral signals; clinical and environmental validation establishes credibility; and surveillance systems aggregate confirmed or high-confidence findings across time and place. The strongest implementation model is therefore not a stand-alone digital or wastewater platform, but an interoperable workflow in which molecular, clinical, environmental, and open-source evidence can be compared and escalated through predefined public-health procedures [27,29,30,31,32,33,34,35,36,37,38,39].

Across the three levels considered in this review, the same principle recurs: AI can increase analytical reach, but its value depends on the quality of upstream laboratory data, the robustness of metagenomic processing, and the context in which outputs are interpreted. The conclusion therefore draws together these interdependent requirements rather than treating metagenomic, clinical, and population applications as separate endpoints.

## 6. Conclusions

AI does not replace conventional virus-detection methods; instead, it acts as a capability multiplier across three levels when laboratories and public-health systems treat reliability and responsible deployment as core requirements [1,8,40]. At the metagenomic level, AI strengthens detection and discovery of novel or highly divergent viruses through pretrained representations and models that can operate on short contigs or under low-homology conditions, as illustrated by ViraLM and LucaProt [3,5]. At the clinical level, mNGS can provide substantial complementary diagnostic value when etiology is uncertain, but clinicians must interpret results within rigorous quality-control frameworks to reduce false positives and avoid incorrect conclusions [1,2]. At the population level, early warning systems can integrate epidemiological, digital, environmental, and wastewater data with AI to improve signal detection and trend forecasting when public-health teams validate and operationalize outputs transparently [27]. The greatest real-world benefit will therefore come from hybrid pipelines that combine molecular data (NGS/mNGS), clinical information, and environmental surveillance. Importantly, the available evidence does not yet support a standardized cost-effectiveness comparison across AI-assisted virus-detection workflows or deployment settings; future implementation studies should therefore report workflow-level costs, resource use, turnaround time, and clinical or public-health impact alongside analytical performance.

## Figures and Tables

**Table 1:** Comparison of representative AI-enabled approaches, with analytical limitations distinguished from operational and deployment issues.

Tool/Application	Input Data	Primary Output	Strengths	Analytical/Algorithmic Limitations	Operational/Deployment Issues
ViraLM [5]	Metagenomic nucleotide contigs	Probability that a contig is viral; candidate prioritization	Uses pretrained genome representations and performs well on short or fragmented contigs	No direct taxonomic or gene-annotation output; performance can be affected by sequence length and domain shift	Prospective clinical validation remains limited; transformer-based analysis can require substantial computational resources
LucaProt [3]	Translated protein sequences plus structure-informed features	Probability that a candidate is a viral RdRP; prioritization of divergent RNA-virus candidates	Combines sequence and structural information and extends discovery beyond weak homology	RdRP-focused rather than a general diagnostic classifier; output still requires downstream taxonomic placement	Structure-informed analysis increases computational demand; routine use requires reproducibility, turnaround-time, and validation evidence
AI-enabled NGS pipeline [4]	NGS reads, assemblies, and variant information	Pathogen detection, validation, and SNP/variant outputs	Integrates several analytical tasks within a reproducible workflow	Performance remains sensitive to upstream quality control, contamination, and reference-database completeness	Requires pipeline maintenance, standardization, external validation, and integration with laboratory quality-management procedures
AI-assisted clinical mNGS [1,2,6,7,9,25,26]	Sequence findings plus technical and clinical metadata	Prioritized and contextualized diagnostic report	Can integrate multiple evidence layers and support patient-level decision-making	Interpretive thresholds vary; contaminants, low-abundance signals, and incidental findings can complicate causal interpretation	Routine adoption requires validated workflows, standardized reporting, suitable turnaround time, trained personnel, and regulatory/accreditation compliance
AI-enabled surveillance systems [8,27,28,29,30,31,32,33,34,35,36,37,38,39]	Genomic, wastewater, epidemiological, digital, and event-based data	Anomaly detection, trend forecasts, or early-warning signals	Integrates heterogeneous population-level signals and can support earlier prioritization of potential threats	Bias, representativeness, data drift, explainability, and false-alert burden can limit reliability	Deployment requires interoperability, privacy and governance safeguards, prospective validation, transparent escalation thresholds, and sustained public-health infrastructure

## Data Availability

No new data were created in this study. The original contributions presented in the study are included in the article.
